# Flourishing Antibacterial Strategies for Osteomyelitis Therapy

**DOI:** 10.1002/advs.202206154

**Published:** 2023-01-30

**Authors:** Xukai Wang, Mingran Zhang, Tongtong Zhu, Qiuhua Wei, Guangyao Liu, Jianxun Ding

**Affiliations:** ^1^ Department of Thoracic Surgery China‐Japan Union Hospital of Jilin University 126 Xiantai Street Changchun 130033 P. R. China; ^2^ Key Laboratory of Polymer Ecomaterials Changchun Institute of Applied Chemistry Chinese Academy of Sciences 5625 Renmin Street Changchun 130022 P. R. China; ^3^ Department of Disinfection and Infection Control Chinese PLA Center for Disease Control and Prevention 20 Dongda Street Beijing 100071 P. R. China

**Keywords:** antibacterial agent, antibacterial mechanism, biomaterial, osteomyelitis therapy, targeted antibacterial strategy

## Abstract

Osteomyelitis is a destructive disease of bone tissue caused by infection with pathogenic microorganisms. Because of the complex and long‐term abnormal conditions, osteomyelitis is one of the refractory diseases in orthopedics. Currently, anti‐infective therapy is the primary modality for osteomyelitis therapy in addition to thorough surgical debridement. However, bacterial resistance has gradually reduced the benefits of traditional antibiotics, and the development of advanced antibacterial agents has received growing attention. This review introduces the main targets of antibacterial agents for treating osteomyelitis, including bacterial cell wall, cell membrane, intracellular macromolecules, and bacterial energy metabolism, focuses on their mechanisms, and predicts prospects for clinical applications.

## Graphical abstract

Various antibacterial agents are developed vigorously for osteomyelitis therapy, including metal‐based antibacterial agents, antibiotics, antibacterial peptides, antibacterial proteins, antibacterial cationic polymers, and so forth, which usually play an anti‐infective role by acting on the targets of cell wall, cell membrane, intracellular macromolecules, and energy metabolism, providing support for the comprehensive treatment of osteomyelitis.

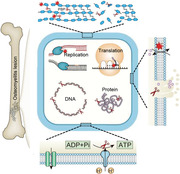

## 1 Introduction

Osteomyelitis is a common orthopedic disease caused by bone tissue infection with pathogenic microorganisms.^[^ ^1^ ^]^ It is generally classified into acute osteomyelitis and chronic osteomyelitis.^[^ ^2^ ^]^ Approximately 10%–30% of acute osteomyelitis cases progress to chronic osteomyelitis.^[^ ^3^ ^]^ According to its pathogenesis, osteomyelitis is divided into hematogenous osteomyelitis, spreading osteomyelitis, and traumatic osteomyelitis.^[^ ^4^ ^]^ Traumatic osteomyelitis is the most common type, accounting for ∼80% of osteomyelitis incidence. Traumatic osteomyelitis is attributable to the entry of pathogenic bacteria into bone tissue through a skin wound or incision,^[^ ^5^ ^]^ which often occurs in open fractures or post‐operative infections. Common pathogens causing osteomyelitis include *Staphylococcus aureus* (*S. aureus*), *Hemolytic streptococcus*, *Pneumococcus*, *Escherichia coli* (*E. coli*), *Pseudomonas aeruginosa*, and others.^[^ ^6^ ^]^ Among them, *S. aureus* is the most common pathogenic bacterium of osteomyelitis, accounting for ∼75% of the bacterial spectrum of osteomyelitis pathogens.^[^ ^7^ ^]^ In addition, poor local bone and soft tissue blood supply in patients with osteomyelitis lead to ischemic sclerosis of the diseased bone, which makes it difficult to achieve locally effective bactericidal concentrations with systemic antibiotics.^[^ ^8^ ^]^ Therefore, osteomyelitis is generally characterized by significant treatment difficulty, long duration, and a high recurrence rate.^[^ ^9^ ^]^ It is one of the most challenging diseases encountered by orthopedic physicians.

At present, anti‐infective therapy is the primary strategy for the treatment of osteomyelitis. The cure rate of acute osteomyelitis reaches up to 80% by early diagnosis combined with sensitive antibiotic therapy.^[^ ^10^ ^]^ However, the local bone loss bone and soft tissue insufficiency in chronic osteomyelitis lead to ischemic sclerosis of the diseased bone. These factors make it challenging to achieve locally effective bactericidal concentrations only with systemic antibiotics.^[^ ^11^ ^]^ Surgical debridement combined with local antibiotics and sustained release systems has been proven to make up for the above deficiencies and significantly improve the cure rate of chronic osteomyelitis.^[^ ^12^ ^]^ Despite advances in surgical and antibiotic therapy, the long‐term recurrence rate of chronic osteomyelitis remains at 20%–30%.^[^ ^13^ ^]^

**Scheme 1:**
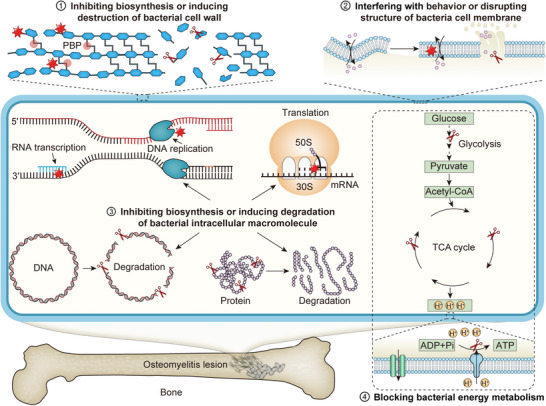
Antibacterial treatment strategies for osteomyelitis.

Antibacterial agents should have a broad‐spectrum effect, excellent antibacterial performance, and limited drug resistance during osteomyelitis treatment.^[^ ^14^ ^]^ Currently, the antibacterial agents commonly used in the treatment of osteomyelitis include metal‐based antibacterial agents, antibiotics, antibacterial peptides, antibacterial enzymes, antibacterial cationic polymers, and so forth. Metal‐based inorganic antibacterial agents have attracted widespread attention due to their broad‐spectrum antibacterial properties, although their biocompatibility is unsatisfactory.^[^ ^15^ ^]^ Antibiotics are the most widely used organic antibacterial agents with high biocompatibility and excellent antibacterial effects.^[^ ^16^ ^]^ However, long‐term systemic administration of high‐dose antibiotics is costly and has severe systemic side effects.^[^ ^17^ ^]^ In recent years, the overuse of antibiotics has led to the emergence of drug‐resistant strains, which have gradually reduced the clinical efficacy of antibiotics.^[^ ^18^ ^]^ Therefore, non‐antibiotic organic antibacterial agents, such as antibacterial peptides and antibacterial cationic polymers, which are not prone to cause bacterial resistance,^[^ ^19^ ^]^ have gradually entered the limelight. However, the biocompatibility and safety of non‐antibiotic organic antibacterial agents are still being investigated. From the perspective of antibacterial strategies, this review summarizes the main targets of various antibacterial agents and focuses on the antibacterial mechanisms, as shown in **Scheme** **1**. By reviewing the targets and mechanisms of different antibacterial agents, we hope to contribute to the comprehensive treatment of osteomyelitis. In addition, this article concludes with an outlook on the future of osteomyelitis treatment.

## 2 Bacterial Cell Wall‐Targeting Strategies

The cell wall is a rigid and elastic structure on the surface of bacteria that protects the bacteria from environmental aggression.^[^ ^20^ ^]^ When the cell wall is disrupted, bacteria lose their natural barrier that plays a role in exchanging material and information with the external environments. In treating osteomyelitis, the bacterial cell wall is one of the targets of antibacterial agents.^[^ ^21^ ^]^ Antibacterial agents mainly exert their antibacterial effect by inhibiting cell wall biosynthesis or disrupting the bacterial cell wall structure.

### 2.1 Inhibition of Biosynthesis

Bacteria are classified into Gram‐positive and Gram‐negative bacteria according to the structure and chemical composition of their cell wall. The main components of bacterial cell wall are peptidoglycan, teichoic acid, lipopolysaccharide, phospholipids, and outer membrane proteins.^[^ ^22^ ^]^ Various antibacterial agents act on the synthesis process of these components, thereby inhibiting cell wall biosynthesis.^[^ ^23^ ^]^

**Figure 1:**
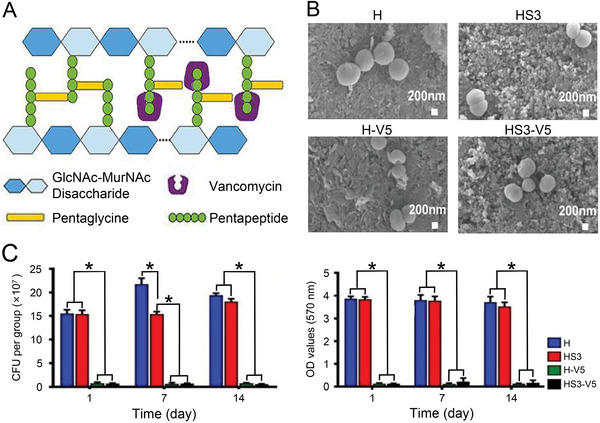
Antibacterial mechanism and effect of VAN. A) Mode of action of VAN in *S. aureus*. Reproduced with permission.^[^ ^26^ ^]^ Copyright 2016, Frontiers Media SA. B) Bacterial morphology of *S. aureus* after 24 h of co‐incubation with different groups of samples. C) Number of colony forming units and bacterial viability of *S. aureus* after 1 day, 7 days, and 14 days of co‐incubation with different groups of samples. Data are shown as mean ± SD (*n* = 3; *P < 0.05). Reproduced with permission.^[^ ^30^ ^]^ Copyright 2021, Elsevier. CFU, colony forming unit; HA, hydroxyapatite; OD, optical density; Si, silicon; VAN, vancomycin.

Peptidoglycan is an essential ingredient for the survival of bacteria by providing a rigid structure for bacteria in a hypotonic environment. Penicillin (PEN)‐binding proteins are enzymes in bacterial cell wall peptidoglycan biosynthesis. PEN and cephalosporin (CEP) are clinically representative *β*‐lactam antibiotics for treating osteomyelitis, which are commonly utilized in the treatment of osteomyelitis due to their penetrating effect on bone tissue.^[^ ^24^ ^]^ *β*‐lactam antibiotics inhibit peptidoglycan synthesis in the bacterial cell wall by acting on PEN‐binding proteins.^[^ ^25^ ^]^ The glycopeptide antibiotic vancomycin (VAN) also exerts its antibacterial effect by inhibiting bacterial cell wall synthesis. The mechanism of action of VAN is to inhibit the formation of a cross bridge between pentapeptide and pentaglycine by binding to the C‐terminal D‐alanine‐D‐alanine residue of pentapeptide (**Figure** **1**A).^[^ ^26^ ^]^ This interferes with the subsequent two‐step enzymatic reaction of trans‐peptide and trans‐glycosylation and ultimately inhibits bacterial cell wall peptidoglycan biosynthesis.^[^ ^27^ ^]^ VAN has been used to treat osteomyelitis due to its excellent antibacterial effect. Ghimire et al. developed an on‐demand VAN release coating using *S. aureus*‐sensitive micrococcal nuclease as a response switch. In vivo experiments showed that this coating eliminated 95% of the bacterial load compared with the control group and effectively prevented the progression of osteomyelitis.^[^ ^28^ ^]^ In a retrospective clinical study, Gelfand and colleagues found that VAN had a cure rate of ∼70% for vertebral osteomyelitis.^[^ ^29^ ^]^

In recent years, the side effects associated with the application of VAN for osteomyelitis therapy have become increasingly significant. Cytotoxicity caused by high local concentrations of VAN results in insufficient bone regeneration. Taking advantage of the positive effect of silicon (Si) on bone regeneration, Xu et al. developed an antibiotic delivery system consisting of hydroxyapatite (HA) and mesoporous bioactive glass coating containing Si and VAN (HS‐V).^[^ ^30^ ^]^ Scanning electron microscopy (SEM) images showed bacterial structural collapse and cell wall damage in the VAN‐loaded H‐V5 and HS3‐V5 groups (Figure 1B). The H‐V5 and HS3‐V5 groups showed a lower number of colony‐forming units and weaker bacterial viability after 1, 7, and 14 days of incubation with different groups of samples (Figure 1C). The antibacterial and bone‐repairing effects of different groups were further verified in a rat model of chronic osteomyelitis. Hematoxylin and eosin (H&E) staining and toluidine blue staining showed that the HS‐V5 group had the slightest inflammation and the most significant bone repair. VAN exerts its antibacterial effect by inhibiting the synthesis of bacterial cell wall.

The bacterial outer membrane is a unique structure of the cell wall of Gram‐negative bacteria.^[31]^ The bacterial outer membrane mainly comprises lipopolysaccharide and phospholipid bilayers with lipoproteins.^[^ ^32^ ^]^ The action of strong barrier and efflux system of the outer membrane greatly diminishes the efficiency of antibiotics in treating osteomyelitis.^[^ ^33^ ^]^ Many antibacterial agents interfere with the biosynthesis of outer membrane by inhibiting lipopolysaccharide synthesis and transport. As a member of the adenosine triphosphate (ATP)‐binding cassette transporter superfamily, the lipopolysaccharide transporter MsbA is an ATP‐dependent lipase mainly responsible for lipopolysaccharide transport. Alexander et al. found that quinoline compounds acted on lipopolysaccharide transporters to interfere with outer membrane biosynthesis and subsequently killed *E. coli*.^[^ ^34^ ^]^ By inhibiting external membrane synthesis, quinoline compounds reduce the resistance of Gram‐negative bacteria to antibacterial agents, thereby improving their therapeutic efficacy for osteomyelitis treatment.

### 2.2 Disruption of Structures

The cell wall is an integral structure for bacterial cell integrity. Its primary function is to maintain the specific shape of bacteria, protecting the bacteria from mechanical or osmotic damage.^[^ ^35^ ^]^ Therefore, antibacterial agents cause bacterial death by disrupting the structural integrity of bacterial cell wall.^[^ ^36^ ^]^

Metal ions and metal/metal oxide nanoparticles exert their antibacterial effects mainly through ion release, contact reaction, and catalytic mechanisms.^[^ ^37^ ^]^ Silver (Ag) preparations are an ancient antibacterial agent. Silver ion (Ag^+^) released by antibacterial agents react with peptidoglycan in the cell wall, resulting in the destruction of basic skeleton structure of bacterial cell wall and exerting an antibacterial effect.^[^ ^38^ ^]^ In addition, silver nanoparticles (AgNPs) with a large specific surface area can be directly adsorbed onto the bacterial cell wall to exert more potent antibacterial effects. Taking advantage of this antibacterial mechanism of Ag, Ye et al. developed a porous sponge composite containing AgNPs by freeze‐drying. This material exhibited excellent antibacterial activity against *S. aureus* and *E. coli*.^[^ ^39^ ^]^ Many metals/metal oxide nanoparticles tend to exhibit enzyme‐like catalytic activity and generate reactive oxygen species (ROS).^[^ ^40^ ^]^ ROS mainly damages the peptidoglycan layer and the lipopolysaccharide layers of bacterial cell wall.^[^ ^41^ ^]^ Due to its high photocatalytic activity, titanium dioxide (TiO_2_) has received extensive attention in the antibacterial field. Hou et al. observed the killing process of *E. coli* by Ag/AgBr/TiO_2_ nanotube array electrode using transmission electron microscopy (TEM). The results showed that ROS attacked the bacterial cell wall early in the photocatalytic reaction. This subsequently led to a gradual rupture of the cell wall and changes in the bacterial morphology. Eventually, the bacteria died due to the disruption of their cell integrity.^[^ ^42^ ^]^

**Figure 2:**
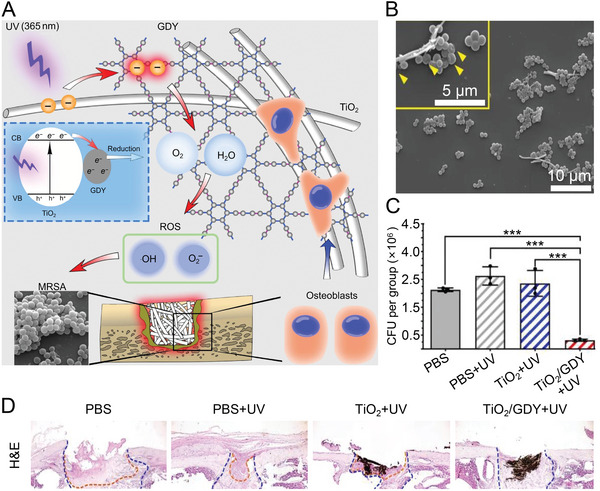
Antibacterial mechanism and effect of ROS generated by photocatalytic reaction. A) Schematic illustration of photocatalytic sterilization and osteogenesis of TiO_2_/GDY. B) SEM images of MRSA after photocatalytic treatment of TiO_2_/GDY nanofiber. In the magnified SEM image, the depressions (yellow arrows) formed after ROS‐induced cell wall perforation can be observed. C) Quantitative analysis of bacterial colonies infecting femurs by UV irradiation with TiO_2_, TiO_2_/GDY, and PBS as controls. Data are shown as mean ± SD (*n* = 3; ***P   <   0.001). D) H&E staining of infected femurs of mice. The orange dashed line indicates the infected area, and between the orange and blue dashed lines is the area of new bone formation. Reproduced with permission.^[^ ^43^ ^]^ Copyright 2020, Springer Nature. CFU, colony forming unit; GDY, graphdiyne; MRSA, methicillin‐resistant *S. aureus*; PBS, phosphate buffered saline; ROS, reactive oxygen species; TiO_2_, titanium dioxide; UV, ultraviolet.

Based on previous research, Wang et al. synthesized graphdiyne‐modified TiO_2_ (TiO_2_/GDY) nanofiber by electrostatic self‐assembly.^[^ ^43^ ^]^ TiO_2_/GDY nanofiber possessed not only long‐lasting antibacterial effects of photocatalytic reaction but also exhibited osteoinductive properties (**Figure** **2**A). ROS generated by the photocatalytic reaction attacked bacterial cell wall, eventually leading to changes in the cell morphology (Figure 2B). Quantitative analysis of bacterial colonies in infected femurs of mice showed that methicillin‐resistant *S. aureus* (MRSA) was reduced by 85% in femurs treated with TiO_2_/GDY+UV, which was superior to other groups (Figure 2C). Moreover, H&E sections of the femur showed that the TiO_2_/GDY nanofiber group had a higher antibacterial effect and osteoinductive ability compared with  other groups (Figure 2D). The results demonstrated that metal‐based inorganic antibacterial agents disrupt the bacterial cell wall through various mechanisms.

In the anti‐infective treatment of osteomyelitis, the bacterial cell wall is also the target of antibacterial peptides.^[^ ^44^ ^]^ Through an electrostatic attraction mechanism, positively charged antibacterial peptides bind to negatively charged substances on the bacterial cell wall, such as lipopolysaccharides and theobromine, which exert antibacterial effects by disrupting or lysing the cell wall.^[^ ^45^ ^]^ Harder et al. found that human µ‐defensin‐3 (HBD‐3) isolated from human lesional psoriatic scales had antibacterial activity against multidrug‐resistant *S. aureus* and VAN‐resistant *Enterococcus faecium*. HBD‐3 caused cell morphology abnormalities in bacteria, leading to cell wall perforation and bacterial death.^[^ ^46^ ^]^ In addition, antibacterial enzymes may also act on the bacterial cell wall to treat osteomyelitis. Lysozyme is a type of alkaline protease that works on cell wall peptidoglycan and exists widely in nature.^[^ ^47^ ^]^ Lysozyme reduces cell wall stability by breaking *β*‐1,4‐glycosidic bonds. The unstable cell wall cannot withstand the osmotic pressure inside and outside the cells, leading to cell rupture and death.^[^ ^48^ ^]^ Xiao et al. developed an antibacterial dressing by loading lysozyme onto polyurethane dressings. The antibacterial dressing containing lysozyme had an excellent antibacterial effect, thus promoting the healing of infected wounds in mice.^[^ ^49^ ^]^ Furthermore, lysostaphin produced by mimetic *S. aureus* disrupts the cell wall of *S. aureus* by hydrolyzing pentaglycine peptide bond bridges on peptidoglycan.^[^ ^50^ ^]^ Johnson et al. designed a four‐armed poly(ethylene glycol)‐maleimide hydrogel loaded with hemolysin to eradicate *S. aureus* in murine osteomyelitis completely.^[^ ^51^ ^]^

As bacterial resistance increases, phage and lytic enzymes have attracted extensive attention as an alternative to antibiotics. The phage‐encoded lytic enzymes hydrolyze peptidoglycan in bacterial cell wall, destroying its structure.^[^ ^52^ ^]^ Wang et al. reported that lytic enzymes derived from phage PSD9 caused leakage of intracellular components by hydrolyzing bacterial cell wall. It showed bactericidal activity against a variety of bacteria.^[^ ^53^ ^]^ Fisher et al. utilized this mechanism and successfully cured osteomyelitis caused by a diabetic foot ulcer in a 60‐year‐old woman using phage.^[^ ^54^ ^]^ Phage therapy could overcome bacterial resistance and may be a potential treatment option for osteomyelitis.

In addition, basic studies have demonstrated that antibacterial cationic polymers adsorb, penetrate, and destroy bacterial cell wall, ultimately leading to bacterial death.^[^ ^55^ ^]^ The synthesized antibacterial cationic polymers mainly include quaternary ammonium compounds, biguanide compounds, quaternary phosphonium salts, and others.^[^ ^56^ ^]^ Wang et al. found that quaternary ammonium salts damaged the bacterial cell wall and cell membrane, resulting in significant leakage of bacterial intracellular substances. They took advantage of quaternary ammonium salts, such as polyurethane coatings, which showed excellent antibacterial activity against *E. coli* and *S. aureus*.^[^ ^57^ ^]^ Corresponding to the synthetic cationic polymer, chitosan is representative of natural cationic polymer. Positively charged chitosan adsorbed negatively charged bacteria and caused rupture of cell wall, resulting in bacterial dissolution and death.^[^ ^58^ ^]^ Moreover, chitosan quaternary ammonium salt is an essential class of chitosan derivatives, which has a higher antibacterial effect because it has a greater positive charge and exhibits stronger adsorption to bacteria. According to Marin et al., chitosan quaternary ammonium salts have a promising future in the antibacterial treatment of osteomyelitis.^[^ ^59^ ^]^ In conclusion, antibacterial cationic polymers have potent contact killing and antibacterial activity, which target the bacterial cell wall for osteomyelitis treatment.^[^ ^60^ ^]^

## 3 Bacterial Cell Membrane‐Targeting Strategies

The bacterial cell membrane is a selective permeability membrane. The cell membrane has critical physiological functions, such as material transport, cell recognition, and cellular immunity.^[^ ^61^ ^]^ In the antibacterial treatment of osteomyelitis, antibacterial agents affect the normal function of cell membrane by interfering with its behaviors. In addition, antibacterial agents also exert antibacterial effects by directly destroying the cell membrane structure.^[^ ^62^ ^]^

### 3.1 Interference of Behaviors and Functions

The bacterial cell membrane is a barrier that maintains the stability of intracellular microenvironments.^[^ ^63^ ^]^ It maintains the concentration balance of different substances inside and outside the cell membrane by controlling the exchange of substances. The normal function of bacterial cell membrane mainly depends on cell membrane behaviors, such as fluidity and selective permeability.^[^ ^64^ ^]^ Fluidity is a structural property of the cell membrane, while selective permeability is a functional property of the cell membrane. Therefore, many antibacterial agents for osteomyelitis exert antibacterial action by interfering with the behaviors and functions of bacterial cell membrane.^[^ ^65^ ^]^

Metal ions encounter negatively charged groups on the cell membrane and bind tightly due to coulomb attraction. This charge interaction between bacterial and metal ions promotes the change of membrane potential state of negative inside and positive outside. The change in the polarization state leads to the interference of normal behaviors and functions of the bacterial cell membrane.^[^ ^66^ ^]^ For example, Ag^+^ increases the permeability of cell membrane by altering the zeta potential at cell surface, which disrupts the balance of cellular material exchange.^[^ ^67^ ^]^ Taking advantage of this mechanism, Mokabber et al. have developed a calcium phosphate coating containing Ag^+^ to enhance the anti‐infective capacity of surgical implants. The results confirmed that the coating reduced bacterial load by 76.1% ± 8.3% through the Ag^+^ leaching mechanism.^[^ ^68^ ^]^ In addition, ROS generated by metal‐based antibacterial agents exerted antibacterial effects by acting on the cell membrane. ROS attacks the unsaturated fatty acids in cell membrane to form lipid peroxidation products, such as malondialdehyde, changing the fluidity and permeability of cell membrane.^[^ ^69^ ^]^ Hong et al. found that copper alloy‐mediated *E. coli* death was mainly mediated by membrane lipid peroxidation.^[^ ^70^ ^]^ Copper ion (Cu^2+^) participate in the Fenton‐like reaction to generate ROS. ROS attacks double bonds in the *E. coli* cell membrane in unsaturated fatty acids. Fatty acid peroxidation changes the physical properties of cell membrane, which affects the fluidity of bacterial cell membrane and causes bacterial death.

Although discovered after metal‐based antibacterial agents,^[^ ^71^ ^]^ antibiotics are currently the most common and effective organic antibacterial agents for treating osteomyelitis. Colistin (COL) and daptomycin (DAP) are used clinically to treat infections caused by multidrug‐resistant bacteria, including refractory osteomyelitis. The common mechanism of these two antibiotics is to interfere with the cell membrane behaviors and functions of bacteria to exert antibacterial effects.^[^ ^72^ ^]^ COL interacts with the bacterial cell membrane, which alters the selective permeability of cell membrane, leading to the efflux of essential substances and demonstrating excellent antibacterial activity. Vallet‐Regí and coworkers employed COL plus Arabic gum to coat moxifloxacin (MOX)‐loaded mesoporous silica nanoparticles. The combination of COL and MOX cleared 90% of bacterial load in rabbit *E. coli* osteomyelitis.^[^ ^73^ ^]^ DAP, the first lipopeptide antibiotic,^[^ ^72b^ ^]^ reacts with negatively charged phosphatidylglycerol and cardiolipin in the cell membrane, resulting in bacterial cell depolarization, altered membrane potential, and impaired exchange of cellular contents.^[^ ^74^ ^]^ Müller et al. utilized different fluorescent lipid probes and conducted an experimental exploration to investigate this mechanism of DAP action further. They finally demonstrated that DAP rearranged the fluid lipid domains of cell membrane and altered the fluidity of bacterial cell membrane.^[^ ^75^ ^]^ In addition, DAP has shown excellent antibacterial efficacy in the clinical treatment of osteomyelitis. Tuon and coworkers retrospectively analyzed 163 patients with internal fixation device‐associated osteomyelitis treated with DAP. The results showed that the clinical cure rate of osteomyelitis after DAP treatment was ∼70%.^[^ ^76^ ^]^ More importantly, DAP also has antibacterial activity against drug‐resistant bacteria. Billups et al. reported a successful case of osteomyelitis treatment with DAP in an eight‐year‐old boy who experienced VAN treatment failure.^[^ ^77^ ^]^

In addition, natural organic antibacterial agents extracted from plants, such as polyphenols and essential oils, are early‐developed antibacterial agents by humans. Hydrophilic polyphenols increase membrane permeability and affect membrane fluidity, thereby affecting the normal function of bacterial cell membrane.^[^ ^78^ ^]^ Yi et al. reported that tea polyphenols acted on the bacterial cell membrane by changing the cell membrane permeability and impairing cell membrane function.^[^ ^79^ ^]^ To further verify the antibacterial effect of tea polyphenols, Mei and coworkers conducted antibacterial experiments using green tea polyphenols‐loaded porous silica nanospheres in a simulated mildly acidic microenvironment of osteomyelitis. The results showed that the nanospheres loaded with green tea polyphenols effectively killed *S aureus*, and its bacterial inhibition rate reached 72% of VAN.^[^ ^80^ ^]^ In addition, Chinese herbal medicines also have antibacterial properties and can be used in treating osteomyelitis. Yang et al. extracted essential oils from traditional Chinese herbal medicines. Conductivity measurements found that crucial oils increased *S. aureus* cell membrane permeability and exerted significant antibacterial effects.^[^ ^81^ ^]^

### 3.2 Destruction of Structures

The barrier function of bacterial cell membrane provides bacteria with a relatively stable internal microenvironment.^[^ ^82^ ^]^ Therefore, once the structural integrity of the bacterial cell membrane is disrupted, the bacteria will die rapidly.^[^ ^83^ ^]^ This mechanism is also exploited to treat osteomyelitis.

Nanomaterials with unique physical structures may disrupt bacterial cell membrane.^[^ ^84^ ^]^ Graphene nanosheets are called nano‐knives with sharp edges. They penetrate the cell membrane and cause physical damage to the cell membrane, leading to leakage of intracellular materials and bacteria death.^[^ ^85^ ^]^ Based on the surface nanostructures of cicadas, Guan et al. developed a coating of TiO_2_ nanorod loaded with polydopamine (PDA) and AgNPs.^[^ ^86^ ^]^ The coating exploited the physical killing effect of TiO_2_ nanorod. SEM images revealed that the TiO_2_ nanorod had an apparent physical puncture effect on *E. coli* and *S. aureus* cell membrane. The nanorod disrupted the bacterial cell membrane without affecting the osteoblasts because the bacteria were smaller in size than osteoblasts.^[^ ^87^ ^]^ Moreover, this difference in volume allows the nanomaterials to exhibit excellent antibacterial effects and biocompatibility. This physical therapy strategy reduces the development of bacterial drug resistance and has a promising therapeutic potential for refractory osteomyelitis.

**Figure 3:**
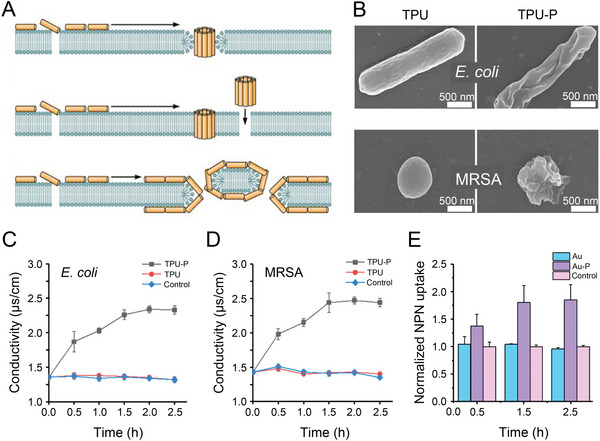
Antibacterial mechanism and effect of antibacterial peptides. A) Membrane dissolution mechanism models of antibacterial peptides of annular pore model, stick model, and carpet model. Reproduced with permission.^[^ ^90c^ ^]^ Copyright 2017, American Society for Microbiology. B) SEM images of *E. coli* and MRSA after 2.5 h of co‐incubation with bare TPU and TPU‐P surfaces. C,D) Changes in electrical conductivity of *E. coli* and MRSA on different surfaces. E) Effects of different surfaces on permeability of *E. coli* cytoplasmic membrane. Reproduced with permission.^[^ ^94^ ^]^ Copyright 2021, Elsevier. Au, gold; Au‐P, peptide polymer modified gold surface; NPN, 1‐N‐phenyl‐naphthylamine; TPU, thermoplastic polyurethane; TPU‐P, peptide polymer‐modified thermoplastic polyurethane.

Antibacterial peptides act on the cell wall and damage the cell membrane structure by interacting with the lipid components.^[^ ^88^ ^]^ The membrane‐targeting mechanism is the most classical and essential mechanism of action for antibacterial peptides. The insertion of hydrophobic residues of antibacterial peptides into cell membrane leads to changes in the membrane structure and the formation of transmembrane ion channels on cell membrane.^[^ ^89^ ^]^ At present, there are many hypothetical models for the membrane permeabilization mechanism of antibacterial peptides,^[^ ^90^ ^]^ such as the barrel plate model, the carpet model, and the ring hole model (**Figure** **3**A). Recently, the membrane‐targeting mechanism of antibacterial peptides in the anti‐infective treatment of osteomyelitis has become a research hotspot. The antibacterial peptides extracted from house flies by Hou et al. exhibited antibacterial activity against various bacteria by disrupting the cell membrane.^[^ ^91^ ^]^ Zarghami et al. developed a Ti composite coating using melittin, and the coating composition included melittin, chitosan, and VAN. Through the synergistic effect of antibiotics and melittin, the coating was able to eradicate VAN‐resistant *S. aureus* within 3 h.^[^ ^92^ ^]^

However, the low stability of antibacterial peptides makes them difficult to prepare in large quantities, and the high cost of synthesis limits their practical application. Antibacterial peptide mimetic polymers solve these shortcomings of antibacterial peptides and have gradually become a research hotspot.^[^ ^93^ ^]^ Liu et al. synthesized an antibacterial peptide mimetic polymer by one‐pot polymerization, which comprised 90% D,L‐lysine and 10% *γ*‐benzyl‐L‐glutamate.^[^ ^94^ ^]^ SEM images showed that the antibacterial peptide‐modified thermoplastic polyurethane (TPU‐P) induced the morphological shrinkage of *E. coli* and MRSA due to the breakdown of bacterial membrane and the outflow of cellular contents (Figure 3B). It was found that TPU‐P increased the electrical conductivity of *E. coli* (Figure 3C) and MRSA (Figure 3D) to ∼2.34 µs cm^−1^, which further proved that the antibacterial peptide polymer caused the leakage of bacterial intracellular substances. Fluorescent probe examination also demonstrated that the antibacterial peptide polymer‐modified gold surface (Au‐P) significantly enhanced the permeability of *E. coli* cell membrane (Figure 3E). To further verify the antibacterial properties and stability of antibacterial peptide polymer, Liu et al. incorporated the prepared antibacterial peptide polymer with polymethyl methacrylate bone cement (PMMA@polymer) and carried out related experiments.^[^ ^95^ ^]^ The PMMA@polymer group killed 99.99% of *S. aureus* within 1 h, as indicated by the in vitro antibacterial test. Furthermore, the PMMA@polymer group exhibited excellent therapeutic effects in a rabbit chronic osteomyelitis model. All the above results suggest that antibacterial peptide polymers exerted antibacterial effects by disrupting the bacterial membrane.

Antibacterial cationic polymers bind to the bacterial cell membrane with positive and negative charges, disrupting the cell membrane structure via the membrane cleavage mechanism.^[^ ^60^ ^]^ Quaternary ammonium salts exert antibacterial effects by destroying the cell membrane structure.^[^ ^96^ ^]^ Wang et al. synthesized polymers containing quaternary ammonium groups at both ends. The antibacterial coating made of this polymer responsively releases quaternary ammonium groups, showing significant antibacterial activity against *S. aureus* and other bacteria.^[^ ^97^ ^]^ Liu et al. evaluated the bactericidal activity of chitosan against *E. coli* and *S. aureus*. The results demonstrated that chitosan disrupted bacterial cell membrane integrity and permeability, resulting in the leakage of cellular contents.^[^ ^98^ ^]^ Based on the antibacterial mechanism of chitosan, Hoque et al. developed a coating using quaternary chitin polymers.^[^ ^99^ ^]^ The coating interacted with the bacterial cell membrane and disrupted the integrity of cell membrane structure, reducing the 3.7 log MRSA burden on the surface of coated medical catheters compared with no‐coating catheters.

## 4 Bacterial Intracellular Macromolecules‐Targeting Strategies

Intracellular macromolecules are important antibacterial targets in treating osteomyelitis,^[^ ^100^ ^]^ mainly including nucleic acids and proteins. Nucleic acid is one of the essential components of organisms, and protein is the main undertaker and executor of life activities. During the anti‐infective treatment of osteomyelitis, various antibacterial agents act by inhibiting the synthesis or inducing the degradation of intracellular macromolecules.^[^ ^101^ ^]^

### 4.1 Inhibition of Biosynthesis

By inhibiting the synthesis of intracellular macromolecules, antibacterial agents hinder bacteria from performing normal life activities, causing their death.^[^ ^102^ ^]^ Precursor substances of nucleic acids, critical enzymes in nucleic acid synthesis, and sites of protein translation are the main targets of action of various antibacterial agents.^[^ ^103^ ^]^

Folic acid is a precursor substance for bacterial nucleic acid synthesis, and bacteria cannot directly utilize folic acid in the extracellular microenvironments due to the poor permeability of bacteria to folic acid. Instead, under the catalysis of dihydrofolate synthase, bacteria use *p*‐aminobenzoic acid, dihydropteridine, glutamic acid, and other substances to synthesize dihydrofolate. Dihydrofolate is converted to tetrahydrofolate by the action of dihydrofolate reductase, which is subsequently involved in the synthesis of nucleic acid precursors.^[^ ^104^ ^]^ Sulfonamide (SUL), structurally similar to *p*‐aminobenzoic acid, competes with dihydrofolate synthase and hinders dihydrofolate synthesis.^[^ ^105^ ^]^ Trimethoprim (TRI) inhibits the synthesis of tetrahydrofolate by reducing the activity of dihydrofolate reductase.^[^ ^106^ ^]^ Therefore, TRI is utilized as a synergist in combination with SUL. SUL has a long history of treating osteomyelitis and has achieved effective clinical therapeutic effects. In a retrospective study, Messina et al. found that 11 children with acute osteomyelitis were cured by trimethoprim‐sulfamethoxazole treatment.^[^ ^107^ ^]^ Heo et al. developed a co‐electrospun scaffold (GE/PLGA‐AgSD) containing various concentrations of silver sulfadiazine (AgSD), which consisted of gelatin (GE) and poly(D,L‐lactide‐*co*‐glycolide) (PLGA) fiber sheets. The experiment showed that the GE/PLGA‐AgSD scaffold had excellent antibacterial effects and biocompatibility, which can be utilized to prevent osteomyelitis.^[^ ^108^ ^]^ Recently, SUL has gradually been withdrawn as a treatment for osteomyelitis due to its weak antibacterial power, susceptibility to resistance, and significant side effects.

Nucleic acid synthesis includes DNA replication and RNA transcription. Many antibiotics currently used for the clinical treatment of osteomyelitis inhibit the nucleic acid synthesis process by acting on the enzymes required. Quinolone antibiotics are the most commonly employed broad‐spectrum antibiotics that inhibit DNA synthesis in clinical practice. Quinolone antibiotics widely applied in clinical treatment include MOX, levofloxacin (LEV), and ciprofloxacin (CIP).^[^ ^109^ ^]^ In Gram‐positive bacteria, the main target of quinolones is DNA gyrase (a subclass of topoisomerase II), which loosens supercoiled DNA and plays an essential role in the initiation of DNA replication. Meanwhile, in Gram‐negative bacteria, the main target is topoisomerase IV, which is involved in unzipping and separating sister chromatids during anaphase. Quinolone antibiotics interfere with the replication and transcription of bacterial chromosomes by interacting with DNA gyrase or topoisomerase VI. Thus, bacteria are inhibited and unable to reproduce, leading to bacterial death.^[^ ^110^ ^]^ RNA polymerase is the most critical enzyme in transcription and an essential antibacterial target.^[^ ^111^ ^]^ The co‐crystal structure formed by rifampicin (RIF) in the cleft near the active center of RNA polymerase restricts the extension of RNA strands in space, which prevents RNA synthesis, thereby having a bactericidal effect.^[^ ^112^ ^]^ At present, orthopedic doctors generally combine RIF with quinolone antibiotics to treat osteomyelitis.^[^ ^113^ ^]^

The ribosome is the site of protein synthesis, and the bacterial ribosome is composed of a small subunit and a large subunit.^[^ ^114^ ^]^ In treating osteomyelitis, the ribosome is the most crucial target of antibacterial agents to inhibit protein synthesis. Gentamicin (GEN) and tetracycline (TET) combine with small subunits to alter or stop bacterial protein synthesis.^[^ ^115^ ^]^ Chloramphenicol (CHL) and erythromycin (ERY) combine with large subunits to inhibit protein synthesis.^[^ ^116^ ^]^ Those are commonly utilized antibiotics in the clinical treatment of osteomyelitis. Oxazolones mainly affect the function of ribosomes, inhibiting protein synthesis by destroying ribosomes.^[^ ^117^ ^]^ At present, linezolid (LIN) is the only oxazolone antibiotic used for clinical treatment. It has the advantages of a high organ distribution rate, intense drug penetration, and high bioavailability to treat Gram‐positive bacterial infections.^[^ ^118^ ^]^ In 2011, the American Academy of Infectious Diseases recommended LIN as a therapeutic drug for osteomyelitis caused by adult MRSA infection.^[^ ^119^ ^]^

Antibacterial peptides also affect cellular physiological activities by inhibiting the synthesis of macromolecules.^[^ ^120^ ^]^ A variety of proline‐rich antibacterial peptides have been identified in Hymenoptera, Diptera, and Hemiptera, which have antibacterial activity against Gram‐negative bacteria by inhibiting the translation of bacterial proteins.^[^ ^121^ ^]^ Bac7_1‐35_ is a proline‐rich antibacterial peptide. Antibacterial peptides also inhibit protein synthesis by acting on the ribosome. Using ribosome co‐precipitation and cross‐linking assays, Mardirossian et al. found that the interaction of Bac7_1‐35_ with ribosomes specifically affected *E. coli* protein synthesis.^[^ ^122^ ^]^ Antibacterial cationic polymers show good antibacterial activity by blocking nucleic acid synthesis or affecting the nucleic acid structure.^[^ ^123^ ^]^ Liu et al. demonstrated that carboxymethylated chitosan not only acted on bacterial cell membrane but also inhibited the synthesis of intracellular macromolecules in *E. coli* by blocking DNA transcription and protein translation.^[^ ^124^ ^]^

### 4.2 Induction of Biodegradation

Intracellular macromolecules are essential components of bacteria and play an important role in the normal execution of bacterial physiological function. Antibacterial agents play an antibacterial role in treating osteomyelitis by inducing the biodegradation of intracellular macromolecules.^[^ ^125^ ^]^

Nitroimidazole antibiotics have shown excellent therapeutic effects on anaerobic bacteria and are commonly used to treat osteomyelitis.^[^ ^126^ ^]^ The main molecules of nitroimidazoles contain the nitro group, which will be reduced to amino group in the absence of oxygen, thus damaging the double helix structure of DNA and eventually inducing DNA biodegradation.^[^ ^127^ ^]^ Currently, nitroimidazole antibiotics commonly used in clinics mainly include metronidazole (MET), tinidazole (TIN), and ornidazole (ORN).^[^ ^128^ ^]^ Inspired by previous work, Jayasree et al. developed a tetracalcium phosphate bone cement containing ORN, which demonstrated obvious antibacterial activity in vitro antibacterial experiments and showed broad application prospects in treating infected bone defects.^[^ ^129^ ^]^

The combination of metal ions with nucleic acids causes denaturation or degradation of the nucleic acids, which leads to sterilization by inhibiting the ability of bacteria to reproduce. In addition, metal ions destroy the spatial conformation of protein molecules or replace the active site of protein, resulting in protein degradation. For example, when Ag^+^ enters the interior of bacteria, it converts DNA into a concentrated state and disrupts the normal folding state of DNA molecules. In addition, Ag^+^ also binds to —SH in the protein and destroys the disulfide bond that maintains the protein structure, inducing protein denaturation.^[^ ^130^ ^]^ Zhong et al. developed a bone morphogenetic protein‐2 (BMP‐2)‐coupled nanosilver‐PLGA composite graft to treat osteomyelitis, utilizing the antibacterial mechanism of Ag^+^.^[^ ^131^ ^]^ Histopathological sections and X‐rays in 12 weeks after surgery confirmed that the femoral osteomyelitis had been cured. The tissue engineering scaffolds not only deliver antibacterial agents but also provide a favorable microenvironment for repairing the attachment of associated cells.^[^ ^132^ ^]^ Therefore, it is commonly utilized in the treatment of osteomyelitis. Lu et al. developed a functional scaffold by loading Ag^+^ and TiO_2_ into nano‐hydroxyapatite/polyamide 66‐based nano‐frame material. This scaffold material exhibited a bactericidal effect on *E. coli* and *S. aureus* in vitro and had an excellent antibacterial impact and biocompatibility in a rabbit osteomyelitis model.^[^ ^133^ ^]^

**Figure 4:**
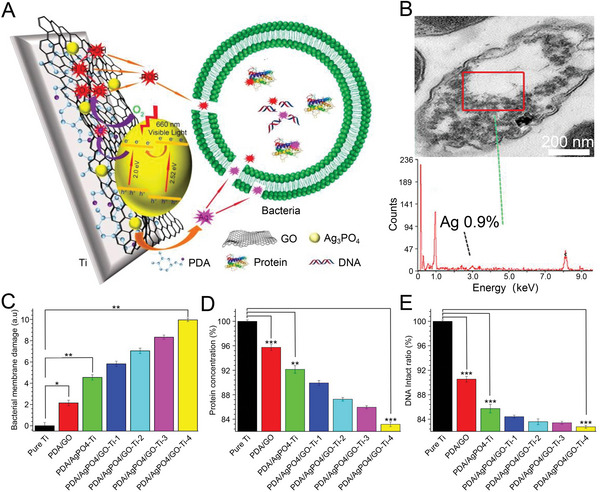
Synergistic antibacterial mechanism and effect of Ag_3_PO_4_/GO coating. A) Ag_3_PO_4_/GO coating destroyed bacterial cell membrane, protein damage, and DNA synergistically with the released Ag^+^ and its generated ROS. B) TEM image and corresponding EDS pattern of bacteria after Ag_3_PO_4_/GO was incubated with *E. coli* bacteria and irradiated with visible light at 660 nm for 10 min. C–E) Bacterial membrane damage histogram, protein concentration histogram, and DNA integrity rate histogram of bacteria after different materials were incubated with *S. aureus* and irradiated with 660 nm visible light for 15 min. Data are shown as mean ± SD (*n* = 3; *P < 0.05, **P < 0.01, ***P < 0.001). Reproduced with permission.^[^ ^136^ ^]^ Copyright 2018, American Chemical Society. GO, graphene oxide; PDA, polydopamine; Ti, titanium.

In addition to acting on bacterial cell wall and membrane, high levels of ROS also induce the degradation of intracellular macromolecules, which is another recognized antibacterial mechanism. ROS damage the antioxidant defense system of bacteria, leading to DNA damage, protein denaturation, lipid peroxidation, and ultimate bacterial death.^[^ ^134^ ^]^ Wei et al. found that microwaves with strong penetrating power effectively treated osteomyelitis in rabbits. Microwave irradiation promoted the release of iron ion (Fe^2+^) from Prussian blue and facilitated their entry into bacteria. A large number of intracellular Fe^2+^ underwent a strong Fenton reaction under the induction of microwave, which led to the production of a large amount of ROS inside the bacteria, resulting in the massive oxidation of glutathione and the final death of bacteria.^[^ ^135^ ^]^ Based on previous studies, Xie et al. designed a polydopamine/Ag_3_PO_4_/graphene oxide (PDA/Ag_3_PO_4_/GO) hybrid coating that exploited the antibacterial mechanism of Ag^+^ and ROS (**Figure** **4**A).^[^ ^136^ ^]^ SEM images showed blank areas in bacterial after *E. coli* was co‐cultured with PDA/Ag_3_PO_4_/GO in vitro, which were caused by the leakage of damaged proteins and DNA. Meanwhile, energy dispersive spectrometer (EDS) results demonstrated that Ag^+^ penetrated bacteria (Figure 4B). When the *S. aureus* colonies were irradiated with 660 nm light for 15 min, the PDA/Ag_3_PO_4_/GO‐Ti‐4 group had the most severe bacterial membrane permeability, and DNA and protein damage (Figure 4C–E) compared with the other groups, and the antibacterial test showed that its antibacterial rate against *S. aureus* was as high as 99.66%. In conclusion, the mechanism of oxidative stress‐induced degradation of intracellular macromolecules provides a new research direction for the treatment of osteomyelitis.

## 5 Bacterial Energy Metabolism‐Targeting Strategies

The energy metabolism of bacteria is the powerhouse of all physiological activities of bacteria and is also a central issue in metabolism.^[^ ^137^ ^]^ Glycolysis, the tricarboxylic acid (TCA) cycle, and the transfer of respiratory electron chains are the most critical steps for bacteria to obtain energy. Bacterial energy metabolism is present throughout the life activities of bacteria. Thus, blocking the energy metabolism step of bacteria is another critical strategy for treating osteomyelitis.^[^ ^138^ ^]^

The energy metabolism process is inseparable from the catalysis of enzymes. Metal inorganic antibacterial agents exert antibacterial properties by inhibiting the activity of enzymes. Phosphofructokinase and glyceraldehyde‐3‐phosphate dehydrogenase are enzymes involved in glycolysis. McEwan and coworkers found that adding zinc ion (Zn^2+^) inhibited the activity of phosphofructokinase and glyceraldehyde‐3‐phosphate dehydrogenase, which ultimately disrupted the glycolysis process in *S. pyogenes*.^[^ ^139^ ^]^ Wang et al. found that Ag^+^ inhibited the activities of isocitrate dehydrogenase in the TCA cycle, resulting in the blockage of TCA cycle.^[^ ^140^ ^]^ Metal‐organic frameworks (MOFs) act as reservoirs for the gradual release of metal ions and have a more effective antibacterial effect than metal nanoparticles.^[^ ^141^ ^]^ Inspired by the antibacterial mechanism of Ag^+^, Zhang et al. synthesized an electrostatically spun fiber mat using silver‐based MOF and poly(lactic acid). The slow release of Ag^+^ within the framework was interfered with *S. aureus* energy metabolism and showed a positive antibacterial effect. In addition, in vivo experiments also demonstrated that the electrostatically spun fiber mat promotes healing of infected wounds in rats.^[^ ^142^ ^]^

**Figure 5:**
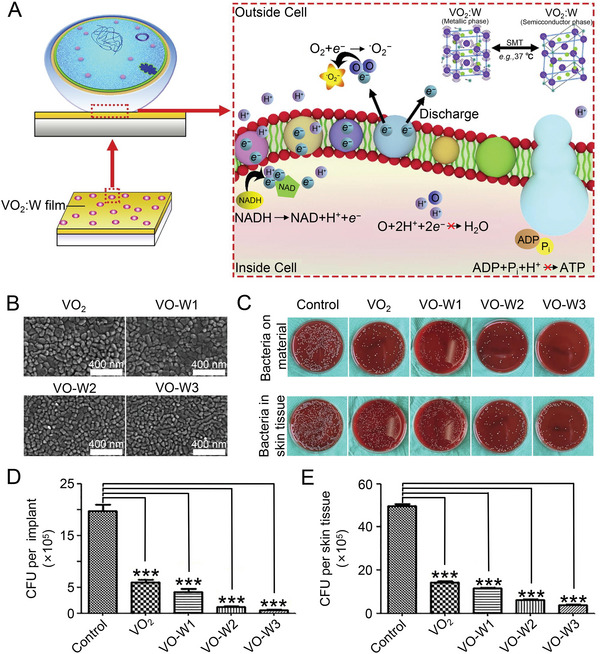
Antibacterial mechanism and effect of VO_2_ films. A) Antibacterial mechanism of conductive VO_2_ films of metallic phase. B) SEM images of VO_2_ films and W‐doped VO_2_ films. C) Representative photographs of re‐cultured bacterial colonies in the material and surrounding soft tissue. D,E) Corresponding quantification of live bacteria in material and surrounding soft tissue. Data are shown as mean ±  SD (*n* = 10; ***P < 0.001). Reproduced with permission.^[^ ^144^ ^]^ Copyright 2019, Elsevier. ADP, adenosine diphosphate; ATP, adenosine triphosphate; CFU, colony forming units; NADH, nicotinamide adenine dinucleotide; VO_2_, vanadium dioxide; W, tungsten.

In addition to acting on enzymes in energy metabolism, metal‐based inorganic antibacterial agents work on other energy metabolism processes. The respiratory electron transport chain is also one of the most critical links in energy metabolism. Metal ions destroy the respiratory electron transport chain, thereby hindering the synthesis of ATP.^[^ ^143^ ^]^ Yeung and coworkers found that metal‐phase vanadium dioxide (VO_2_) films in contact with bacteria caused electron destructive extraction of respiratory chain transmembrane protein complexes,^[^ ^144^ ^]^ resulting in blocked bacterial ATP synthesis (**Figure** **5**A). Tungsten may impart temperature‐responsive conductivity to VO_2_ films. Inspired by this, they prepared VO_2_ films and three groups of tungsten‐containing VO_2_ films (VO‐W), whose SEM images are shown in Figure 5B, and further verified the antibacterial activity by relevant experiments. In vitro antibacterial experiments revealed that the VO_2_ group exhibited antibacterial activity compared to the control group, while the antibacterial effect of VO_2_ films containing tungsten was further enhanced. The diffusion plate method was used to quantify the microbial burden in implants and soft tissues around implants in a rat infection model to verify its antibacterial effect further. Representative photographs of bacterial colonies in each group are shown in Figure 5C. Corresponding quantification of bacteria in implants and soft tissues (Figure 5D,E) showed that all implant groups exhibited higher antibacterial activity than the control group. Moreover, the VO‐W3 group showed minor bacterial residues at (0.57 ± 0.15) × 10^5^ and (3.85 ± 0.24) × 10^5^, respectively. The results demonstrated that metal‐based inorganic antibacterial agents inhibited bacterial energy metabolism from treating bone infections by disrupting electron transfer in the respiratory transport chain.

Furthermore, metal ion‐induced acid–base imbalance in the extracellular microenvironments affects bacterial energy metabolic processes. Ji et al. found that the alkaline microenvironments induced by magnesium oxide (MgO) films consumed protons outside bacteria, disrupted the transport of respiratory electron chains, and eventually inhibited ATP synthesis.^[^ ^145^ ^]^ Inspired by previous studies, Cai and coworkers utilized layer‐by‐layer (LBL) technology to spin‐coat gelatin and chitin onto levofloxacin (Levo)‐loaded zeolitic imidazolate framework‐8 (ZIF‐8@Levo) nanoparticles and developed ZIF‐8@Levo/LBL coating.^[^ ^146^ ^]^ The coating exerted an antibacterial effect by blocking bacterial energy metabolism during femoral osteomyelitis treatment in rats. Different samples were incubated with *E. coli* and *S. aureus*, and the results showed that the ATP levels of *E. coli* and *S. aureus* were significantly decreased in the ZIF‐8@Levo/LBL group. The same effects were also observed in the quantitative analysis of antibacterial rates, in which the antibacterial rates of ZIF‐8@Levo/LBL against *E. coli* and *S. aureus* were as high as 88.5% and 86.4%, respectively. H&E and Masson staining of rat femoral osteomyelitis samples showed that the ZIF‐8@Levo/LBL group had the least inflammatory infiltration and the most significant bone formation. In conclusion, the bacterial energy metabolism process is another critical antibacterial target of inorganic antibacterial agents.

In addition to inorganic antibacterial agents that act on bacterial energy metabolism, natural organic antibacterial agents also exert antibacterial effects by inhibiting the activity of glycolytic and TCA cycle enzymes. Hexokinase and pyruvate kinase are enzymes involved in glycolysis. Black pepper chloroform extract inhibited glycolysis in *E. coli* and *S. aureus* by inhibiting the activities of hexokinase and pyruvate kinase.^[^ ^147^ ^]^ Essential oils, as a natural antibacterial substance, have been used in traditional medicine to exert antibacterial effects by disrupting the TCA cycle. Lin and coworkers found that oregano essential oils inhibited the activities of citrate synthase, isocitrate dehydrogenase, and *α*‐ketoglutarate dehydrogenase during the TCA cycle, thereby blocking the energy metabolism of MRSA.^[^ ^148^ ^]^ Inspired by the antibacterial principles of essential oils, Badea et al. found that hydroxyapatite nanoparticles encapsulated with peppermint essential oils exhibited excellent antibacterial activity against *E. coli* and *S. aureus*. Natural organic antibacterial agents offer a solution for the treatment of osteomyelitis.^[^ ^149^ ^]^

## 6 Multi‐Target Combination Strategies

In recent years, the incidence of osteomyelitis caused by drug‐resistant bacteria has gradually increased, making treating osteomyelitis increasingly tricky.^[^ ^150^ ^]^ The drawbacks of traditional single‐target antibacterial strategies have steadily emerged. Thus, there is an urgent need to discover a more advanced antibacterial approach. The research hotspot of antibacterial strategies for osteomyelitis has changed from “single‐targe” to “multi‐targe”.^[^ ^151^ ^]^ Multi‐target antibacterial techniques improve therapeutic efficacy, reduce the dosage and cytotoxicity of single antibacterial agents, and delay the emergence of bacterial resistance.^[^ ^152^ ^]^

**Figure 6:**
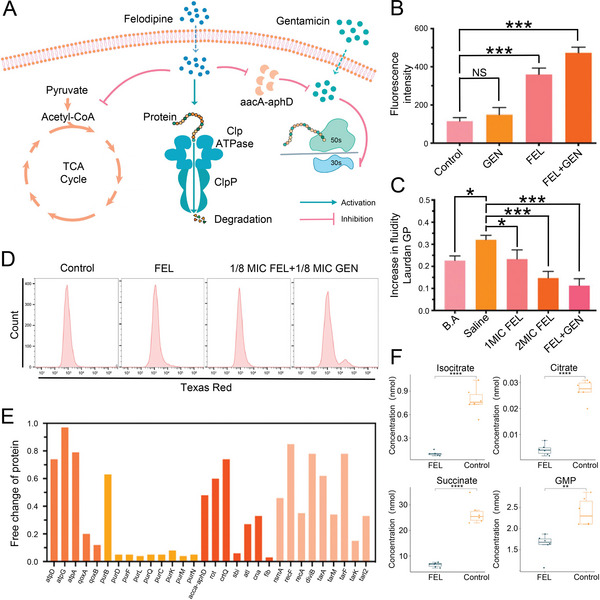
Synergistic antibacterial mechanism and effect of FEL combined with GEN. A) Schematic illustration of antibacterial mechanism of FEL combined with GEN. B) Spectrophotometers monitored fluorescence intensity to assess bacterial cell membrane permeability. Data are shown as mean ± S.D (*n* = 3; NS, no significance; ***P < 0.001). C) Assessment of cell membrane fluidity in MRSA by Laurdan generalized polarization. Data are shown as mean ±  S.D (*n* = 3; *P < 0.05, ***P < 0.001). D) GEN uptake was measured by flow cytometry after incubation with 1 MIC FEL, 1/8 MIC FEL+1/8 MIC GEN. E) PRM examined the expression levels of proteins related to energy metabolism, bacterial resistance, and biofilm formation. F) Detection of energy metabolites associated with the TCA cycle of MRSA after 8 h of incubation with FEL. Data are shown as mean ±  S.D (*n* = 6; **P < 0.01, ****P < 0.0001). Reproduced with permission.^[^ ^153^ ^]^ Copyright 2022, Elsevier. aacA‐aphD, aminoglycoside resistance protein; B.A, benzyl alcohol; ClpP, caseinolytic protease P; FEL, felodipine; GEN, gentamicin; GMP, guanosine monophosphate; Laurdan GP, Laurdan generalized polarization; MIC, minimal inhibitory concentrations; TCA, tricarboxylic acid.

Currently, clinical treatment of osteomyelitis usually combines antibiotics with different mechanisms of action to achieve multi‐target therapy. For example, the combination of glycopeptide and aminoglycoside antibiotics exerts antibacterial effects by inhibiting the synthesis of bacterial cell walls and intracellular macromolecules. In addition to combining antibiotics with different antibacterial targets, researchers have explored other combination approaches. For example, combining antibiotics with non‐antibiotic drugs has also achieved better antibacterial effects. Felodipine (FEL) is a dihydropyridine calcium channel blocker, and Zhang et al. found that combining FEL with aminoglycosides led to a synergistic antibacterial effect.^[^ ^153^ ^]^ FEL exerted antibacterial effects by inhibiting the energy metabolism of bacteria or binding to caseinolytic protease P (ClpP) to induce the degradation of proteins associated with biofilm formation. In addition, FEL also enhances the antibacterial activity of GEN by inhibiting the expression of aminoglycoside resistance‐associated protein (aacA‐aphD) (**Figure** **6**A). The combination of these two drugs decreased the permeability of MRSA cell membrane (Figure 6B) and increased the fluidity of cell membrane (Figure 6C). Flow cytometry assays further confirmed that low doses of FEL increased the uptake of GEN by MRSA (Figure 6D). Broth microdilution checkerboard assay showed that only 1/8 minimal inhibitory concentrations (MIC) of FEL combined with 1/8 MIC of GEN inhibited the proliferation of bacteria. Parallel response monitoring (PRM) examined 30 candidate target proteins and found that FEL inhibited the expression of proteins associated with bacterial energy metabolism, aminoglycoside resistance (aacA‐aphD), and biofilm formation (Figure 6E). Targeted metabonomics showed that MRSA treated with FEL showed lower metabolites related to the TCA cycle (Figure 6F). In addition, histopathological analysis of mouse femur osteomyelitis showed that FEL combined with GEN had the least inflammatory cell infiltration and the lowest bacterial load compared with the single‐agent group. In conclusion, multi‐target combination strategies improve the efficacy of osteomyelitis and reduce the dose of antibacterial agents.^[^ ^154^ ^]^

Antibiotics are also taken in combination with antibacterial peptides to treat osteomyelitis.^[^ ^155^ ^]^ The combination of antibacterial peptides and antibiotics dramatically improves the efficacies of antibiotics. The main reason is that antibacterial peptides change the cell membrane permeability, thereby causing a more significant amount of antibiotics to penetrate the cell and bind to intracellular targets. Shamova et al. found that the combination of antibacterial peptide LL‐37 and GEN accelerated bacterial cell membrane damage. Furthermore, the increase in membrane permeability further enhances the effect of antibiotics and reduces the side effects of high concentrations of antibiotics.^[^ ^156^ ^]^ Phages and antibiotics have different bactericidal mechanisms, and combining these two antibacterial agents produces a synergistic therapeutic effect on the target bacteria, significantly improving the antibacterial effect. In support of these claims, Jacobs and coworkers found that phages increased the susceptibility of multidrug‐resistant *P. aeruginosa* to ceftazidime and ciprofloxacin by 63% and 81%, respectively.^[^ ^157^ ^]^

Different from single‐target antibiotics, multi‐target antibiotics refer to a single drug with multiple antibacterial targets. With the increasing problem of bacterial resistance, there is growing interest in developing multi‐targeted antibiotics. Multi‐target antibiotics were developed in the early 2000s, and the US Food and Drug Administration (FDA) has approved 14 of them over the past 20 years.^[^ ^158^ ^]^ Studies have shown that multi‐target antibiotics are less likely to develop drug resistance than single‐target antibiotics. For example, the development of fluoroquinolone resistance is slow because it simultaneously acts on two targets, DNA gyrase and topoisomerase IV.^[^ ^159^ ^]^

In addition to developing new antibiotics, many essential pieces of research have been carried out on the multi‐target antibacterial effects of metal ions and metal/metal oxide nanoparticles. However, the antibacterial selectivity and biocompatibility of metals remain a great challenge, and there is still much room for clinical translation in treating osteomyelitis. Furthermore, multi‐target antibacterial polymers have also attracted attention due to their high antibacterial efficacy, broad antibacterial spectrum, and drug resistance. Bai et al. synthesized a double‐selective polymer, which selectively destroyed the bacterial cell membrane and simultaneously targets and binds bacterial DNA, showing bactericidal effects on various bacteria.^[^ ^160^ ^]^ Chen et al. developed amphiphilic oligomers with hydrophobic carbon chains and cationic groups and found that the oligomers selectively damaged bacterial cell membrane and DNA. The antibacterial mechanism of this amphiphilic mode exhibits superior antibacterial properties,^[^ ^161^ ^]^ bringing a broad application space for the treatment of osteomyelitis.

## 7 Conclusions and Perspectives

Currently, clinical treatment of osteomyelitis relies on anti‐infective therapy and surgery. However, the increasing prevalence of drug‐resistant bacteria has rendered the treatment of osteomyelitis more difficult. New antibacterial agents are constantly being developed with innovations and advances in medical technology. The antibacterial strategies of multiple antibacterial agents for treating osteomyelitis need to be summarized. Therefore, we reviewed the different classes of antibacterial agents used for treating osteomyelitis and outlined the targets and mechanisms of antibacterial agents, as shown in **Table** **1**.

**Table 1:** Summary of antibacterial targets and mechanisms of antibacterial agents on osteomyelitis

Antibacterial target	Antibacterial mechanism	Types of antibacterial agent	Specific example	Reference	Year
Bacterial cell wall	Inhibition of biosynthesis	Antibiotic	PEN CEP	[24] [25]	2009 2020
			VAN	[27]	2018
	Disruption of structure	Metal‐based antibacterial agent	Ag^+^	[38]	2008
			AgNPs	[39]	2015
			Ag/AgBr/TiO_2_ nanotube array electrode	[42]	2012
			TiO_2_/GDY nanofiber	[43]	2020
		Antibacterial peptide	HBD‐3	[46]	2001
		Antibacterial enzyme	Lysozyme	[47]	2019
			Lysostaphin	[50]	2014
			Lytic enzyme	[52]	2021
		Antibacterial cationic polymer	Quaternary ammonium compound, biguanide compound, and quaternary phosphonium salt	[56]	2005 2009 2015
Bacterial cell membrane	Interference of behaviors and functions	Metal‐based antibacterial agent	Ag‐containing calcium phosphate coating	[68]	2020
			Copper alloy	[70]	2012
		Antibiotic	COL DAP	[72]	2020 2021
		Natural plant source organic antibacterial agent	Tea polyphenol	[79]	2013
			Essential oil	[81]	2021
	Inhibition of biosynthesis	Antibacterial inorganic nanomaterial	Graphene nanosheet	[85]	2016
			TiO_2_ nanorod	[86]	2019
		Antibacterial peptide	Melittin	[92]	2021
		Antibacterial peptide mimetic polymer	P(D,L‐Lys_90_‐*co*‐BLG_1__0_)	[94]	2021
		Antibacterial cationic polymer	Quaternary ammonium salt	[96]	2004
			Quaternary chitin polymer	[99]	2016
Bacterial intracellular macromolecules	Inhibition of biosynthesis	Antibiotic	SUL TRI	[105] [106]	2014 2020
			MOX LEV CIP	[109]	2020 2017 2005
			RIF	[112]	2005
			GEN TET	[115]	2001 2000
			CHL ERY	[116]	2016 2005 2014
			LIN	[118]	2011 2001
		Antibacterial peptide	Bac7_1‐35_	[122]	2014
		Antibacterial cationic polymer	Carboxymethylated chitosan	[124]	2000
	Induction of biodegradation	Antibiotic	MET	[128]	2003
			TIN		2016
			ORN	[129]	2018
		Metal‐based antibacterial agent	Ag^+^	[130]	2016
			BMP‐2 coupled‐nanosilver‐PLGA composite graft	[131]	2010
			Fe^2+^	[135]	2021
			PDA/Ag_3_PO_4_/GO coating	[136]	2018
Bacterial energy metabolism	Blocking bacterial energy metabolism	Metal‐based antibacterial agent	Zn^2+^	[139]	2015
			Ag^+^	[140]	2019
			MgO thin film	[145]	2020 2022
			ZIF‐8@Levo/LBL coating	[146]	2020
		Natural organic antibacterial agent	Black pepper chloroform extract	[147]	2018
			Essential oil	[148]	2019

Abbreviation: Ag^+^, silver ion; Ag_3_PO_4_, silver phosphate; AgBr, silver bromide; AgNPs, Ag nanoparticles; BMP‐2, bone morphogenetic protein‐2; CEP, cephalosporin; CHL, chloramphenicol; CIP, ciprofloxacin; COL, colistin; DAP, daptomycin; ERY, erythromycin; Fe^2+^, iron ion; GDY, graphdiyne; GEN, gentamicin; GO, graphene oxide; HBD‐3, human µ‐defensin‐3; LBL, layer‐by‐layer technology; LEV, levofloxacin; LIN, linezolid; MET, metronidazole; MgO, magnesium oxide; MOX, moxifloxacin; ORN, ornidazole; PDA, polydopamine; P(D,L‐Lys_90_‐*co*‐BLG_10_), antibacterial peptide mimetic polymer of 90% D,L‐lysine and 10% γ‐benzyl‐L‐glutamate; PEN, penicillin; PLGA, poly(D,L‐lactide‐*co*‐glycolide); ‐ RIF, rifampicin; SUL, sulfonamide; TET, tetracycline; TIN, tinidazole; TiO_2_, titanium dioxide; TRI, trimethoprim; VAN, vancomycin; ZIF‐8, zeolitic imidazolate framework‐8; Zn^2+^, zinc ion.

Metal‐based antibacterial agents, antibiotics, antibacterial peptides, antibacterial enzymes, and antibacterial cationic polymers are commonly used antibacterial agents for osteomyelitis. These antibacterial agents mainly act on the cell wall, cell membrane, intracellular macromolecules, and energy metabolism to exert anti‐infective effects. The cell wall maintains the intrinsic bacterial morphology and protects bacteria from invasion. Antibacterial agents kill bacteria by targeting the bacterial cell wall, causing the bacteria to lose their protective barrier. Second, the bacterial cell membrane, which has the function of protection, material exchange, and information transfer, is also an essential antibacterial target. Antibacterial agents exert antibacterial effects by interfering with the behaviors and functions of cell membrane or disrupting the cell membrane structure. In addition, antibacterial agents disrupt bacterial physiological activities by inhibiting the synthesis or inducing the degradation of macromolecules. Finally, antibacterial agents also exert antibacterial effects by blocking bacterial energy supply. These antibacterial targets and mechanisms are essential elements of the current antibacterial strategy for osteomyelitis.

Although most of the current research on antibacterial therapy for osteomyelitis is still in the initial stages, multi‐target combination antibacterial strategies have already shown advantages in treating osteomyelitis. Multi‐target combination strategies reduce the dose of antibacterial agents and avoid the generation of bacterial drug resistance. Meanwhile, as the research on antibacterial targets and mechanisms continues to advance, the long‐term effects and potential biological toxicity of multi‐target combination therapy strategies will also be addressed. Multi‐target combination therapy strategies will become the mainstream strategy for osteomyelitis treatment. It is believed that the vigorous development of medical technology will provide an ideal solution for osteomyelitis treatment in the future.

## Conflict of Interest

The authors declare no conflict of interest.
